# Systemic Beta-Hydroxybutyrate Affects BDNF and Autophagy into the Retina of Diabetic Mice

**DOI:** 10.3390/ijms231710184

**Published:** 2022-09-05

**Authors:** Maria Consiglia Trotta, Carlo Gesualdo, Hildegard Herman, Sami Gharbia, Cornel Balta, Caterina Claudia Lepre, Marina Russo, Annalisa Itro, Giovanbattista D’Amico, Luisa Peluso, Iacopo Panarese, Gorizio Pieretti, Giuseppe Ferraro, Francesca Simonelli, Michele D’Amico, Settimio Rossi, Anca Hermenean

**Affiliations:** 1 Department of Experimental Medicine, University of Campania “Luigi Vanvitelli”, Via Santa Maria di Costantinopoli 16, 80138 Naples, Italy; 2 Eye Clinic, Multidisciplinary Department of Medical, Surgical and Dental Sciences, University of Campania “Luigi Vanvitelli”, Via Luigi de Crecchio 6, 80138 Naples, Italy; 3 “Aurel Ardelean” Institute of Life Sciences, Vasile Goldis Western University of Arad, 86 Revolutiei Av., 310414 Arad, Romania; 4 PhD Course in Translational Medicine (XXXV Cycle), Department of Experimental Medicine, University of Campania “Luigi Vanvitelli”, Via Santa Maria di Costantinopoli 16, 80138 Naples, Italy; 5 Department of Mental and Physical Health and Preventive Medicine, University of Campania “Luigi Vanvitelli”, Largo Madonna Delle Grazie 1, 80138 Naples, Italy

**Keywords:** diabetic retinopathy, hydroxycarboxylic acid receptor 2, beta-hydroxybutyrate, brain-derived neurotrophic factor, autophagy, microglia

## Abstract

**Background:** Diabetic retinopathy (DR) is a neurovascular disease, characterized by a deficiency of brain-derived neurotrophic factor (BDNF), a regulator of autophagy. Beta-hydroxybutyrate (BHB), previously reported as a protective agent in DR, has been associated with BDNF promotion. Here, we investigated whether systemic BHB affects the retinal levels of BDNF and local autophagy in diabetic mice with retinopathy; **Methods:** C57BL/6J mice were administered with intraperitoneal (i.p.) streptozotocin (STZ) (75 mg/kg) injection to develop diabetes. After 2 weeks, they received i.p. injections of BHB (25–50–100 mg/kg) twice a week for 10 weeks. Retinal samples were collected in order to perform immunofluorescence, Western blotting, and ELISA analysis; **Results:** BHB 50 mg/kg and 100 mg/kg significantly improved retinal BDNF levels (*p* < 0.01) in diabetic mice. This improvement was negatively associated with autophagosome–lysosome formations (marked by LC3B and ATG14) and to higher levels of connexin 43 (*p* < 0.01), a marker of cell integrity. Moreover, BHB administration significantly reduced M1 microglial activation and autophagy (*p* < 0.01); **Conclusions:** The systemic administration of BHB in mice with DR improves the retinal levels of BDNF, with the consequent reduction of the abnormal microglial autophagy. This leads to retinal cell safety through connexin 43 restoration.

## 1. Introduction

First described as a solely microvascular disease, diabetic retinopathy (DR) is now a well-known pathological condition in which vascular alterations are closely related to neuronal degeneration and mitochondrial damage of retinal cells induced by chronic inflammation [1]. In this regard, the neuroprotective brain-derived neurotrophic factor (BDNF) has recently emerged as a potential target in retinal disorder pathogenesis, due to its beneficial effects on retinal neuronal layers [2]. Indeed, its dysregulation has been reported in preclinical and clinical studies, evidencing decreased BDNF levels in a rat model of early retinal neuropathy induced by diabetes [3] as well as in serum and aqueous humor of diabetic patients before DR clinical signs [4]. 

Worthy of note, BDNF deficiency in different DR cell culture models was shown to contribute to abnormally increased autophagy [5], evidenced by enhanced microtubule-associated protein light chain 3 B (LC3B) expression [6]. This is a marker of autophagosome formation [7] strictly involved in the degradation of connexin 43 (Cnx43) [8], a gap junction reduced in DR and associated to retinal vascular cell death [9,10,11].

Therefore, a BDNF up-regulation could be a therapeutic option for DR by restoring the correct autophagic response into the retina, leading to retinal cell safety through Cnx43 restoration. To this regard, BDNF levels seem to be induced in the central nervous system (CNS) by beta-hydroxybutyrate (BHB) [12,13,14], a ketone body capable of activating the hydroxycarboxylic acid receptor 2 (HCA_2_), with consequent reduction of the NOD-like receptor protein 3 (NLRP3) inflammasome activity and preservation of the retina from DR damage in a mouse model [9]. To our knowledge, no previous studies examined the expression levels of BDNF in the retina and its involvement in the local autophagic response.

The present study firstly aims to investigate the effects of exogenous BHB (25–50–100 mg/kg) on retinal BDNF and consequent autophagy in diabetic C57BL/6J mice. Then, since the retina expresses different types of neurons (e.g., ganglion and amacrine neurons) and glia (e.g., astrocytes, Muller cells) [15] and BHB inhibits microglia activation [16,17,18], the specific relation between BHB-BDNF and retinal microglial autophagy, not yet fully elucidated but crucial for neuron function and survival [19], will be analyzed in the same experimental setting. 

## 2. Results

### 2.1. BHB Restores Retinal BDNF Levels and Activity 

As previously seen in other studies by this group, retinas from diabetic mice (75 mg/kg streptozotocin—STZ group) were characterized by damage at several levels: starting from 10-weeks of diabetes, retinas exhibited autophagy and apoptotic nuclei, with ultrastructural changes in retinal pigmented epithelial (RPE) cells, the inner nuclear layer (INL), and the outer nuclear layer (ONL). Particularly, INL was characterized by dark irregular nuclei, disrupted processes in Müller cells, activated microglia, swollen mitochondria, and reduced BDNF staining [20], while ONL exhibited apoptotic cells and low Cnx43 expression [9]. Furthermore, retinal vessels showed an increased tortuosity starting from 12-week of diabetes, with the presence of irregular retinal vessel caliber, microaneurysms low occluding, and high vascular endothelial growth factor (VEGF) levels after 16 weeks of diabetes [21].

**Figure 1:**
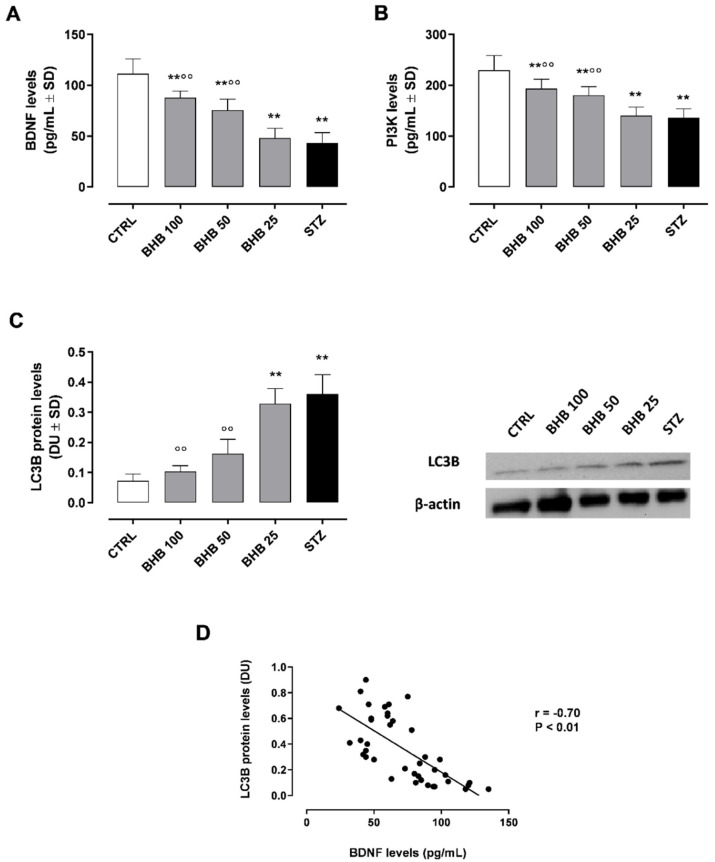
Retinal levels of BDNF (**A**), PI3K (**B**), LC3B (**C**), and strength of association between BDNF and LC3B (**D**) in non-diabetic mice (CTRL), diabetic mice (STZ), and diabetic mice treated with BHB 25 mg/kg (BHB 25), 50 mg/kg (BHB 50), and 100 mg/kg (BHB 100). BDNF (pg/mL) and LC3B (densitometric units, DU) levels are expressed as mean ± SD of N = 8 retinas per group. ** *p* < 0.01 vs. CTRL; °° *p* < 0.01 vs. STZ.

In line with previous evidence [20], here it is shown a significant reduction of BDNF levels (43 ± 10 pg/mL, *p* < 0.01) in diabetic retina compared to non-diabetic mice (CTRL group; 111 ± 14 pg/mL) (Figure 1A). 

BDNF levels were significantly restored by intraperitoneal (i.p.) BHB at the doses of 50 mg/kg (BHB 50 group; 88 ± 7 pg/mL, *p* < 0.01 vs. STZ) and 100 mg/kg (BHB 100 group; 75 ± 11 pg/mL, *p* < 0.01 vs. STZ) (Figure 1A). To monitor retinal BDNF activity, the levels of phosphotylinosital 3 kinase (PI3K), one of the proteins induced following BDNF activation [22], have been dosed. These were significantly reduced in STZ mice (136 ± 18 pg/mL, *p* < 0.01 vs. CTRL) and markedly improved in diabetic mice by BHB 50 mg/kg up to 180 ± 17 pg/mL (*p* < 0.01 vs. STZ) and by 100 mg/kg BHB up to 193 ± 18 pg/mL (*p* < 0.01 vs. STZ) (Figure 1B). 

### 2.2. BHB Reduces the Diabetes-Induced Autophagy through BDNF

The analysis of retinal LC3B protein levels, indicating autophagosome formation [7], showed a significant up-regulation of retinal LC3B levels, expressed as densitometric units (DU), in STZ mice (0.36 ± 0.06 DU, *p* < 0.01 vs. CTRL) (Figure 1C and Figure S1). Daily systemic administration of the autophagy activator MG-132 (10 µg/kg i.p.) over two weeks+STZ did not change the retinal LC3B levels with respect to STZ alone (Figure S2A). In both BHB 50 and BHB 100 groups, the increase in retinal BDNF levels was paralleled by a significant decrement of LC3B protein expression (0.16 ± 0.04 and 0.10 ± 0.02 DU, respectively, both *p* < 0.01 vs. STZ) (Figure 1C). Pearson correlation analysis confirmed a significant negative association between BDNF and LC3B in retinal samples (r = −0.70, *p* < 0.01) (Figure 1D). 

### 2.3. BDNF Upregulation by BHB Reduces the Autophagosome–Lysosome Formation in Diabetic Retina 

**Figure 2:**
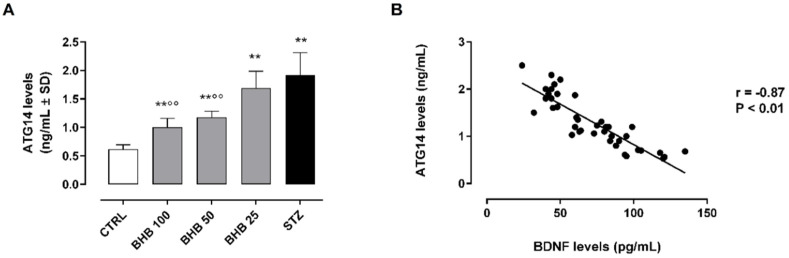
Retinal ATG14 levels (**A**) and their strength of association with BDNF (**B**) in non-diabetic mice (CTRL), diabetic mice (STZ), and diabetic mice treated with BHB 25 mg/kg (BHB 25), 50 mg/kg (BHB 50), and 100 mg/kg (BHB 100). ATG14 levels (ng/mL) are expressed as mean ± SD of N = 8 retinas per group. ** *p* < 0.01 vs. CTRL; °° *p* < 0.01 vs. STZ.

The retinal levels of the autophagy-related 14 protein (ATG14, or ATG14L, or beclin-1-associated autophagy-related key regulator), a specific marker of the autophagosome–lysosome fusion [23], were significantly increased in diabetic retina (2.0 ± 0.3 ng/mL, *p* < 0.01 vs. CTRL) (Figure 2A). The first three days of systemic administration of the lysosome inhibitor bafilomicin A1 at a dose of 0.3 mg/kg/day i.p.+STZ did not change the retinal levels of ATG14 with respect to STZ alone (Figure S2B). The BHB treatment (50 and 100 mg/kg) induced a marked reduction of ATG14 levels (BHB 50: 1.2 ± 0.1 ng/mL and BHB 100: 1.0 ± 0.1 ng/mL, both *p* < 0.01 vs. STZ), significantly inversely correlated with retinal BDNF levels (r = −0.87, *p* < 0.01) (Figure 2A,B). 

### 2.4. BHB Improves Retinal Gap Junctions in Diabetic Retina trough the Regulation of Autophagosome–Lysosome Formation

**Figure 3:**
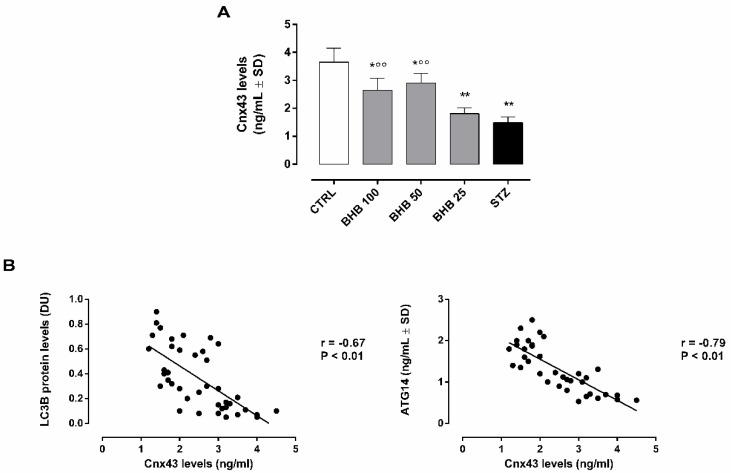
Retinal Cnx43 levels (**A**) and their strength of association with LC3B and ATG14 (**B**) in non-diabetic mice (CTRL), diabetic mice (STZ), and diabetic mice treated with BHB 25 mg/kg (BHB 25), 50 mg/kg (BHB 50), and 100 mg/kg (BHB 100). Cnx43 levels (ng/mL) are expressed as mean ± SD of N = 8 retinas per group. * *p* < 0.05 and ** *p* < 0.01 vs. CTRL; °° *p* < 0.01 vs. STZ.

As expected, Cnx43 retinal levels were downregulated in diabetic retina (1.5 ± 0.2 ng/mL, *p* < 0.01 vs. STZ), while they were promoted by BHB administration (BHB 50: 2.9 ± 0.4 ng/mL and BHB 100: 2.7 ± 0.4 ng/mL, both *p* < 0.01 vs. STZ) (Figure 3A). 

The Cnx43 increment showed a significant negative correlation with both LC3B (r = −0.67, *p* < 0.01) and ATG14 (r = −0.79, *p* < 0.01) (Figure 3B). 

### 2.5. BHB Reduces M1 Microglial Activation in Diabetic Retina

**Figure 4:**
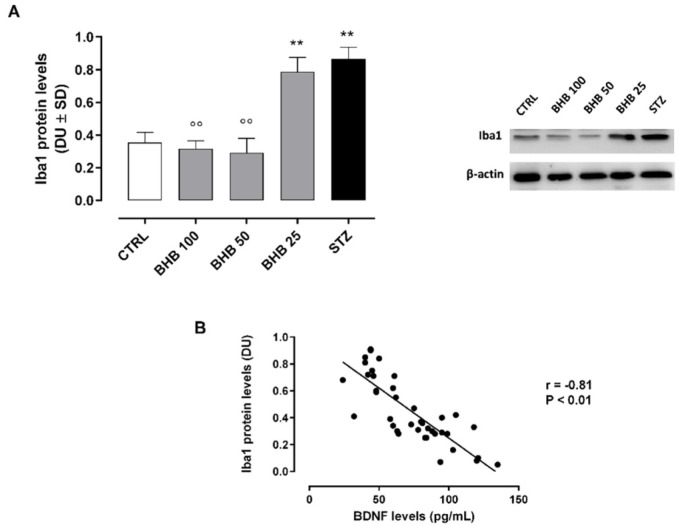
Retinal Iba1 levels (**A**) and their strength of association with BDNF (**B**) in non-diabetic mice (CTRL), diabetic mice (STZ), and diabetic mice treated with BHB 25 mg/kg (BHB 25), 50 mg/kg (BHB 50), and 100 mg/kg (BHB 100). Iba1 levels (densitometric units, DU) are expressed as mean ± SD of N = 8 retinas per group. ** *p* < 0.01 vs. CTRL; °° *p* < 0.01 vs. STZ.

The reduction of retinal BDNF in diabetic retina was paralleled by significantly increased retinal levels of ionized calcium-binding adapter molecule 1 (Iba1), indicating a higher M1 microglial activation (0.86 ± 0.07 DU, *p* < 0.01 vs. CTRL) (Figure 4A and Figure S1).

These were reduced in diabetic animals administered with BHB 50 mg/kg (0.29 ± 0.9 DU, *p* < 0.01 vs. STZ) and 100 mg/kg (0.32 ± 0.05 DU, *p* < 0.01 vs. STZ) (Figure 4A), showing a significant negative association between Iba1 and BDNF retinal levels (r = −0.83, *p* < 0.01) (Figure 4B). 

### 2.6. BHB Decreases Retinal Microglial Autophagy Dysregulated by Diabetes

**Figure 5:**
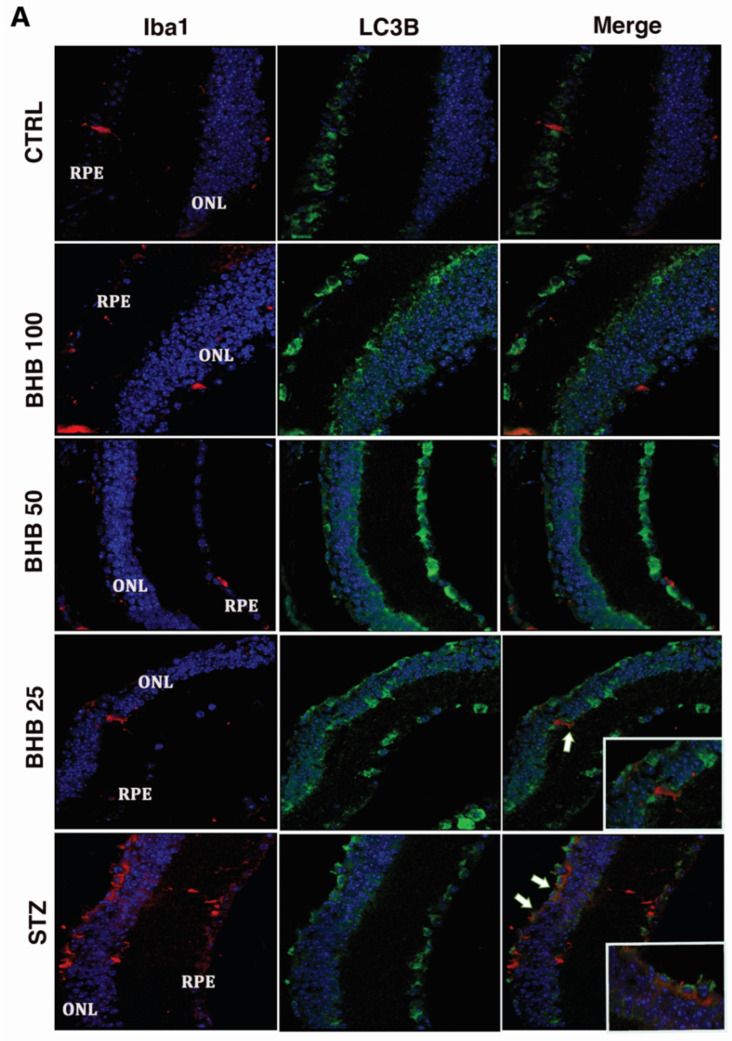

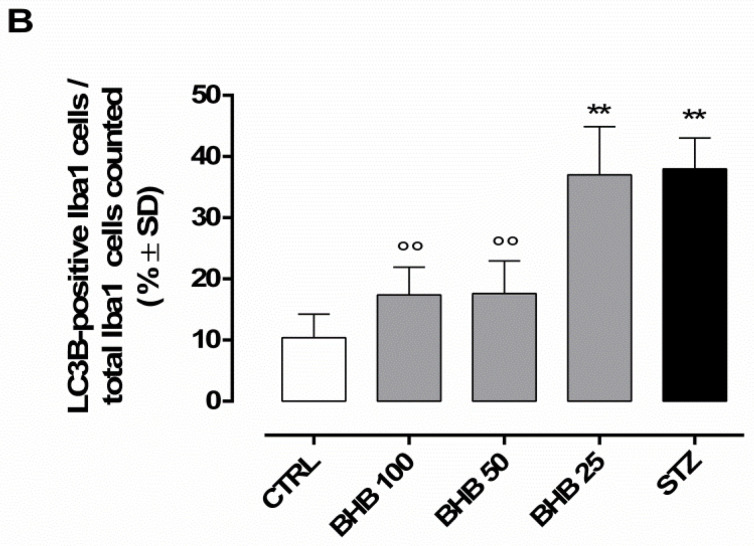
Detection of LC3b immunolabeling in Iba1-positive microglia (**A**) and relative graph (**B**) in non-diabetic mice (CTRL), diabetic mice (STZ), and diabetic mice treated with BHB 25 mg/kg (BHB 25), 50 mg/kg (BHB 50), and 100 mg/kg (BHB 100). (**A**) Sagittal retina sections of visible retinal pigment epithelium (RPE) and outer nuclear layer (ONL), showing the higher co-localization (arrow) of Iba1 (red) and LC3B (green) in the ONL of STZ and BHB 25 groups. Magnification ×63. (**B**) Graph expressing the percentage (%) of LC3B-positive Iba1 cells on total Iba1 cells counted. The results are reported as mean ± SD of N = 8 retinas per group. ** *p* < 0.01 vs. CTRL; °° *p* < 0.01 vs. STZ.

The analysis of the LC3B-positive Iba1 microglia in our experimental setting showed the highest number of Iba1 cells (red staining) expressing LC3B (green staining) in diabetic retinas, particularly in ONL (38 ± 5%, *p* < 0.01 vs. CTRL) (Figure 5A,B). 

BHB treatment at 50 mg/kg and 100 mg/kg markedly reduced the co-localization between Iba1 and LC3B, showing a reduction of M1 microglial autophagy in ONL (BHB 50: 17.6 ± 5%, *p* < 0.01 vs. STZ; BHB 100: 17.4 ± 5%, *p* < 0.01 vs. STZ) (Figure 5A,B). 

### 2.7. HCA_2_ Blocking by Pertussis Toxin (PTX) Abolishes BHB Effects on BDNF and Cnx43

**Figure 6:**
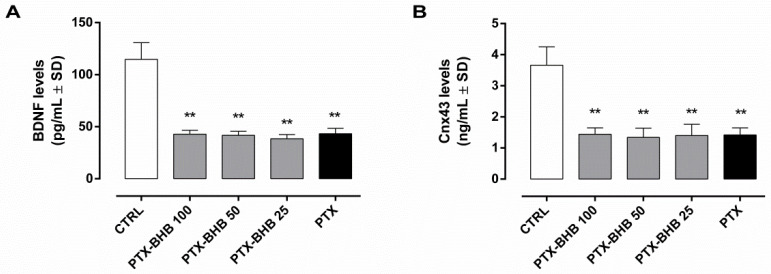
Retinal levels of BDNF (**A**) and Cnx43 (**B**) in non-diabetic mice (CTRL), diabetic mice treated with PTX (1 µg/100 µL) (PTX) and diabetic mice treated with both PTX and BHB 25 mg/kg (PTX-BHB 25), 50 mg/kg (PTX-BHB 50), and 100 mg/kg (PTX-BHB 100). BDNF (pg/mL) and Cnx43 (ng/mL) levels are expressed as mean ± SD of N = 8 retinas per group. ** *p* < 0.01 vs. CTRL.

The blocking of the HCA_2_ receptor by PTX (1 µg) i.p. administration to diabetic mice markedly reduced the retinal BDNF (43 ± 5 pg/mL, *p* < 0.01 vs. CTRL), also when combined with BHB 25 mg/kg (38 ± 4 pg/mL), 50 mg/kg (42 ± 4 pg/mL), and 100 mg/kg (43 ± 4 pg/mL) in diabetic animals (Figure 6A). 

Similarly, retinal Cnx43 levels were decreased by PTX in diabetic animals (1.4 ± 0.2 ng/mL, *p* < 0.01 vs. CTRL) and were not modified in BHB diabetic animals receiving PTX (BHB 25: 1.4 ± 0.4 ng/mL; BHB 50: 1.3 ± 0.3 ng/mL; BHB 100: 1.4 ± 0.2 ng/mL) (Figure 6B). 

## 3. Discussion

A previous study from this group showed that the receptor for HCA_2_ is overexpressed in the retina following diabetes and its activation with the systemically administered compound BHB can reduce the NLRP3 inflammasome activity and reduce retinal damage [9]. BHB is a ketone body formed during the ketogenic diet recently accredited for beneficial actions in several pathologies [24], exerted by physiologically activating the HCA_2_, a Gi/Go protein-coupled receptor [25]. However, the quantity of BHB may be insufficient to fully activate an overexpressed HCA_2_ receptor, as in diabetic retinal damage [9]. In this case, the STZ-induced diabetic mouse model, being not associated to BHB serum elevation, was useful to increase retinal BHB levels through systemic administration, due to the higher permeability of the damaged retinal barrier [9]. 

Here, it is further elucidated the role of BHB in the protection from DR, by evidencing its association with retinal BDNF levels and activity. This was in line with previous evidence reporting the induction of specific BDNF promoters by BHB in hippocampus neuron cell lines [12,13,14,26,27] and a link between serum BHB and proBDNF in healthy older adults [28]. However, our results suggested a BDNF promotion by BHB in diabetic mice retinas, which has not been previously highlighted by other studies. Indeed, diabetic mice from our experimental setting showed decreased BDNF retinal levels, confirming the previous results reported in the retina of diabetic rodents [3,20] and in the serum of patients with non-proliferative diabetic retinopathy (NPDR) [4,29]. Interestingly, the systemic administration of BHB in diabetic mice almost restored BDNF levels towards the levels of non-diabetic mice. This was in line with the increased plasma BDNF levels reported in hyperglycemic patients assuming BHB [30]. Besides the promotion of BDNF levels, we also found a BHB-induced BDNF activity in diabetic retina. Particularly, in accordance with previous studies [18,31], BHB was able to increase the levels of PI3K, an enzyme induced after BDNF activation and involved in the regulation of neural plasticity, stress resistance, and cell survival [22]. 

As is well known, BDNF is a key mediator in retinal homeostasis, involved in the attenuation of retinal cell death induced by hypoxia [32], in the protection of the inner retinal layer during the aging process [33], in the survival of retinal ganglion cells (RCG) in glaucoma experimental models [34,35,36,37,38,39], and in the preservation of mitochondrial function during the degeneration of retinal photoreceptors [7]. Particularly, in these cells, BDNF deficiency is associated with an increase in LC3-mediated autophagosome formations [7], contributing to the exacerbation of retinal autophagy. 

Abnormal retinal autophagy has recently emerged as a frequent event in neurological disorders [40] and in preclinical and clinical DR settings [5,41,42] and has been indicated as a novel therapeutic target for DR. Particularly, in this in vivo DR model, the marker of autophagosome formation LC3 was found upregulated in diabetic retinas, as well as the marker of autophagosome–lysosome formation ATG14. While LC3 upregulation in diabetic retina and its association with BDNF deficiency was in line with previous preclinical studies [7,38,43,44,45,46,47,48,49,50,51], it is the first time that increased ATG14 levels have been reported to be associated with diabetes-induced retinal damage, although the upregulation of other ATG-related proteins has been widely demonstrated in DR models [44,45,51,52,53]. Therefore, reducing abnormal autophagy may help the cells of the retina to keep their structure and function. Since BHB seems to impact on autophagy dynamics in cortical neurons [54] and BDNF can act as a suppressor of neuronal and retinal autophagy [7,43,55], this study also aimed to investigate if the upregulation of retinal BDNF induced by BHB could impact on the retinal autophagy induced by diabetes. Our results confirmed the reduction of autophagolysosome formation previously described after BHB [54,56,57], but also pointed out that in diabetic retina this process is mediated by the BHB-induced BDNF, exerting a reduction of retinal LC3 and ATG14. Since LC3 and ATG14 are two actors implicated in Cnx43 degradation [8] and Cnx43 has been considered a marker of retinal damage during DR [9,10,11], we here investigated a possible modulation of Cnx43 expression by BHB through the BDNF-LC3-ATG14 pathway. In line with previous evidence [9,58], our results show an up-regulation of Cnx43 retinal levels in diabetic animals treated with BHB, favoring the protective retinal Cnx43-linked process, such as the promotion of retinal vascular homeostasis [10]. We confirmed this pathway involving the BHB-induced BDNF levels reducing the autophagosome–lysosome fusion and ultimately increasing Cnx43, by the intraperitoneal administration of an HCA_2_ antagonist in diabetic animals receiving BHB: the HCA_2_ blocking abolished the increment of both BDNF and Cnx43 retinal levels. 

An important feature of the overall autophagy process is microglial autophagy. This is a functional step crucial in preventing neurodegeneration, by promoting the degradation and the clearance of specific components, which could be toxic for neurons’ survival [19,59,60]. However, when dysregulated, this process could impair the neuron functions, by impacting lipid peroxidation [61].

Worthy of note, BHB has been previously reported to reduce the activation of microglial cells [16,17,18]. These represent the guardian of the insults to the CNS. Upon activation, microglia respond to damage and promote the repair of injury and restoration of homeostasis [62]. However, in the wake of continuous insults, such as it is in diabetes and retinopathy, activated microglia secrete multiple pro-inflammatory cytokines and mediators that overwhelm the reparative capacity of microglia and are detrimental to nearby retinal neurons [63]. Among the two known phenotypes of microglia, M1 has a pro-inflammatory role when activated for a long time, while M2 has a pro-resolving role, by favoring tissue repair [64]. Therefore, controlling M1 microglia activation could be helpful for DR management. In this context, here we showed that BHB can reduce the expression of M1 microglia markers within the ONL of diabetic mice, meaning less activation of these cells following BHB. 

Along with this, after treatment with BHB, we here report a reduction of the dysregulated microglial autophagy. Here we show for the first time that in diabetic retina, LC3B increased into the microglia, seen by immunohistochemical co-localization of Iba1 and LC3B. This was reduced after the treatments of diabetic mice with BHB with respect to diabetes alone, by indicating a reduction of retinal neurons dysfunction. 

Therefore, taking together all these results, our opinion is that the systemic administration of BHB could be a potential therapeutic approach aimed to restore BDNF retinal levels and to reduce its downstream pathways, such as autophagosome–lysosome formation. However, considering the significant sex differences showed by C57BL/6J mice in response to BHB [65], further studies will be needed to investigate a different outcome of BHB treatment in female diabetic mice.

## 4. Materials and Methods

### 4.1. Animals and Experimental Procedures

The experimental protocol here described was approved by the Institutional Ethical Committee for Research of the “Vasile Goldis” Western University of Arad (number 29/17.05.2017) and all efforts were made to minimize animal number and suffering, according to the recommendations of the National Sanitary Veterinary and Food Safety Authority of Romania. 

A single i.p. injection of STZ (75 mg/kg) (U-9889; Santa Cruz Biotechnology, Dallas, TX, USA), freshly dissolved in 10 mM citrate buffer (pH 4.5) [60], was used to induce type-2 diabetes in 7 to 10 week-old male C57BL6J mice, housed in a controlled environment (21–23 °C, 12–12 h light–dark cycle and humidity 55–60%) and fed on a standard chow pellet diet and water ad libitum. 

Particularly, C57BL/6J mice were randomized in animals receiving a single injection of 10 mM citrate buffer (CTRL group, N = 12) and animals receiving a single i.p. injection of STZ 75 mg/kg and developing diabetes (STZ group, N = 55). Fasting blood glucose levels higher than 250 mg/dl on 2 consecutive weeks (Glucometer Elite XL; Bayer Corp., Elkhart, IN, USA) from the STZ injection were verified to assess diabetes development and were confirmed in 48 animals. At this time point, diabetic mice were further randomized in animals receiving i.p. BHB injections at the doses of 25 mg/kg (BHB 25 group, N = 12), 50 mg/kg (BHB 50 group, N = 12) and 100 mg/kg (BHB 100, N = 12) [9]. These doses were previously intraperitoneally tested in diabetic mice, to assess the development of diabetic ketoacidosis (DKA) after their administration: following the highest BHB dose (100 mg/kg), the BHB levels detected in sera were around 1 mmol/L [9], far below the values considered as diagnostic criteria for DKA diagnosis in humans (3.0–3.8 mmol/L) [66].

All BHB injections were administered twice a week for 10 weeks, a period needed to develop high glucose-induced retinal alterations according to our previous experience [9,21]. During this period, fasting blood glucose levels were intermittently monitored and were not affected by BHB administration (Table S1). The remaining untreated STZ mice (N = 12) served as diabetic control group. At the end of the 10 weeks, mice were euthanized by cervical dislocation to dissect retina tissues, as previously described [21]. After the removal of retina samples from C57BL/6J mice, they were prepared for immunofluorescence or biochemical analysis.

To further investigate the influence of STZ-diabetes on the autophagic response, three additional groups of mice (N = 4 each) were considered. In the first group (MG-132), STZ (75 mg/kg, single injection) and the autophagy activator MG-132 (SML1135; Sigma-Aldrich, Milan, Italy) [67] were administered i.p. at the same time. Particularly, MG-132 was daily injected at the dose of 10 µg/kg for 2 weeks [68], after being dissolved in 0.0025 μg/mL dimethyl sulfoxide (DMSO) and then diluted with saline. In the second group (BafA1), STZ (75 mg/kg, single injection) and the lysosome inhibitor bafilomycin A1 (B1793; Sigma-Aldrich, Milan, Italy) [69,70] were administered i.p. contemporaneously. Bafilomycin A1, solubilized in 0.5% DMSO at a concentration of 0.4 mg/100 µL and brought at the final concentration with saline, was daily administered at a dose of 0.3 mg/kg for the first 3 days [71], to avoid its potential disruption of proteasomal and vesicular dynamics [72]. In the third group (Veh), mice were administered with STZ and 0.5% DMSO as vehicle. After two weeks, fasting blood glucose levels higher than 250 mg/dl were verified in all animals, before euthanasia and retina dissection.

### 4.2. Enzyme-Linked Immunosorbent Assay (ELISA) 

BDNF, PI3K, ATG14, and Cnx43 levels were detected in retina samples (N = 8) by using the Mouse BDNF Elisa kit (E-EL-M0203; Elabscience, Houston, TX, USA), the Mouse Phosphotylinosital 3 kinase, PI3K ELISA Kit (CSB-E08419m; Cusabio, Houston, TX, USA), the Mouse Beclin 1-associated autophagy-related key regulator (ATG14) ELISA Kit (abx503511; abbexa, Cambridge, UK), and the Mouse Connexin 43 ELISA Kit (MBS729401; MyBiosource, San Diego, CA, USA), according to the manufacturer’s protocols. 

### 4.3. Immunofluorescence 

Retina sections were deparaffinated in Bond Dewax Solution (Leica Biosystems Inc., Buffalo Grove, IL, USA) and rehydrated in alcohol solutions. To retrieve antigens, Epitope Retrieval Solution (Leica Biosystems Inc., Buffalo Grove, IL, USA) was used at 60 °C overnight, before slides were blocked in a solution of phosphate-buffered saline (PBS) with 2% bovine serum albumin (BSA). Then, slides were incubated for 2 hrs at room temperature with rat monoclonal Iba1 (ab-283346; Abcam PLC., Cambridge, UK; 1:100) and rabbit polyclonal LC3B (ab48394; Abcam; PLC., Cambridge, UK; 1:200) antibodies, both diluted in primary antibody diluting buffer (Bio-Optica, Milano, Italy). After the PBS washing steps, AlexaFluor 488 anti-rabbit IgG (a-11034; Invitrogen, Waltham, MA, USA) and AlexaFluor 546 anti-rat IgG (a-11081; Invitrogen, Waltham, MA, USA) secondary antibodies, both diluted in PBS 1:400, were applied in the dark for 30 min at room temperature. Slides were then washed again with PBS to counterstain cell nuclei with 1 μg/mL 4′,6-diamidino-2-phenylindole (DAPI) (Sigma-Aldrich, St Louis, MO, USA) and mounted the stained slides with CC/Mount aqueous mounting medium (Sigma-Aldrich, St Louis, MO, USA), before their examination with a Leica TSC SP8 laser scanning confocal microscope [73].

### 4.4. Protein Isolation and Quantization 

Retinas were immediately frozen in liquid nitrogen and stored at −80 °C for the subsequent protein isolation. Tissue homogenization was performed in RIPA buffer (R0278; Sigma-Aldrich, Milan, Italy) supplemented with protease and phosphatase inhibitor cocktails (11873580001; Roche, Monza, Italy and 88667; Thermo Fischer, Waltham, MA, USA). Acid nucleic contaminations were removed by centrifuging tissue lysates at 12,000 rpm for 10 min at 4 °C [73]. The protein content in the supernatant fractions was assessed by using the Bio-Rad protein assay (500-0006; Bio-Rad Laboratories, Milan, Italy).

### 4.5. Western Blotting

Western Blotting was performed as previously described [73]. Briefly, protein separation from retinas (N = 5) were performed on a 12% sodium dodecyl sulfate polyacrylamide gel electrophoresis (SDS–PAGE). This was followed by protein electrotransfer to polyvinylidene difluoride (PVDF) membrane (IPFL10100; Merck Millipore, Milan, Italy). Blocking of membranes was performed with a 5% non-fat dry milk/Tris Buffered Saline (TBS) solution for 1 h. Then, membranes were incubated overnight at 4 °C with the following primary antibodies, all diluted in 3% blocking solution: anti-Iba1 (3 µg/mL; ab5076; Abcam; PLC., Cambridge, UK); anti-LC3B (1 µg/mL; ab48394; Abcam; PLC., Cambridge, UK), and β-actin (1:1000; sc47778; Santa Cruz Biotechnology, Dallas, TX, USA). Blots were then incubated with horseradish peroxidase-conjugated secondary anti-rabbit, anti-goat, and anti-mouse antibodies (all 1:10000; respectively, sc-2004, sc-2020, and sc-2005; Santa Cruz Biotechnology, Dallas, TX, USA), for 1 h at room temperature. Iba1 and LC3B immunoreactive bands were detected by using an enhanced chemiluminescence system (35055; Thermo Fisher, Waltham, MA, USA). Then, these were quantized with VisionWorks Life Science Image Acquisition and Analysis software (UVP, Upland, CA, USA), normalized with β-actin protein levels and expressed as densitometric units (DU). 

### 4.6. In Vivo Proof of Concept

To confirm the action of BHB on BDNF and Cnx43 levels via HCA_2_, we set an additional experimental setting to block HCA_2_ activity. Since there are no specific antagonists of these receptor, we used PTX, which disrupts all G-coupled signals [74]. The following animal groups (N = 4 mice per group) were considered: diabetic animals receiving a single i.p. injection of PTX 1 µg in 100 µL of PBS [75], after two consecutive weeks of diabetes (PTX group); PTX animals receiving i.p. BHB injections at the doses of 25 mg/kg (PTX-BHB 25 group), 50 mg/kg (PTX-BHB 50 group), and 100 mg/kg (PTX-BHB 100) twice a week for 10 weeks. Then, animals were euthanized, and retina samples (N = 8 per group) were prepared for BDNF and Cnx43 levels. 

### 4.7. Statistical Analysis 

Results are expressed as the mean ± standard deviation (SD) of N = 8 retinas for each type of analysis. Statistical significance was obtained by one-way analyses of variance (ANOVA), followed by Tukey’s multiple comparison test. Pearson correlation analysis was carried out to evaluate the strength of association between pairs of variables. For both ANOVA and Pearson correlation, a P-value less than 0.05 was considered significant to reject the null hypothesis.

## Data Availability

All data relevant to the study are included within the article and its Supplementary Materials.

## Supplementary Materials

The following are available online at https://www.mdpi.com/article/10.3390/ijms231710184/s1.
