# Supplementary material for: Systemic Beta-Hydroxybutyrate Affects BDNF and Autophagy into the Retina of Diabetic Mice
Source: Int J Mol Sci. 2022 Sep 5;23(17):10184. doi: 10.3390/ijms231710184 (PMC9455989; doi:10.3390/ijms231710184)
Supplement: Supplementary file 1 [file ijms-23-10184-s001.zip › ijms-1863762-supplementary.pdf]

International Journal of *Molecular Sciences*

MDPI

Systemic Beta-Hydroxybutyrate Affects BDNF and Autophagy into the Retina of Diabetic Mice

Maria Consiglia Trotta [1,†], Carlo Gesualdo [2,†], Hildegard Herman [3], Sami Gharbia [3], Cornel Balta [3], Caterina Claudia Lepre [1], Marina Russo [1], Annalisa Itro [4], Giovanbattista D'Amico [1], Luisa Peluso [1], Iacopo Panarese [5], Gorizio Pieretti [2], Giuseppe Ferraro [2], Francesca Simonelli [2], Michele D'Amico [1,‡], Settimio Rossi [2,*,‡] and Anca Hermenean [3,‡]

1 Department of Experimental Medicine, University of Campania "Luigi Vanvitelli", Via Santa Maria di Costantinopoli 16, 80138 Naples, Italy
2 Eye Clinic, Multidisciplinary Department of Medical, Surgical and Dental Sciences, University of Campania "Luigi Vanvitelli", Via Luigi de Crecchio 6, 80138 Naples, Italy
3 "Aurel Ardelean" Institute of Life Sciences, Vasile Goldis Western University of Arad, 86 Revolutiei Av., 310414 Arad, Romania
4 PhD Course in Translational Medicine (XXXV Cycle), Department of Experimental Medicine, University of Campania "Luigi Vanvitelli", Via Santa Maria di Costantinopoli 16, 80138 Naples, Italy
5 Department of Mental and Physical Health and Preventive Medicine, University of Campania "Luigi Vanvitelli", Largo Madonna delle Grazie 1, 80138 Naples, Italy
* Correspondence: settimio.rossi@unicampania.it
† These authors contributed equally to this work.
‡ These authors contributed equally to this work.

Supplementary Materials

Table S1. Fasting blood glucose levels monitored during the 10-week BHB treatments. ** P < 0.01 vs CTRL.

	Blood glucose levels (mg/dl)					
	**Diabetes development**	**Week 2**	**Week 4**	**Week 6**	**Week 8**	**Week 10**
**CTRL**	95 ± 15	92 ± 11	100 ± 5	87 ± 8	94 ± 10	98 ± 6
**BHB 100**	285 ± 23 **	291 ± 28 **	284 ± 20 **	288 ± 17 **	275 ± 18 **	274 ± 13 **
**BHB 50**	292 ± 28 **	294 ± 21 **	289 ± 24 **	291 ± 25 **	287 ± 20 **	280 ± 19 **
**BHB 25**	294 ± 24 **	297 ± 17 **	291 ± 24 **	293 ± 15 **	288 ± 24 **	281 ± 25 **
**STZ**	290 ± 22 **	293 ± 27 **	280 ± 17 **	284 ± 19 **	271 ± 12 **	265 ± 10 **

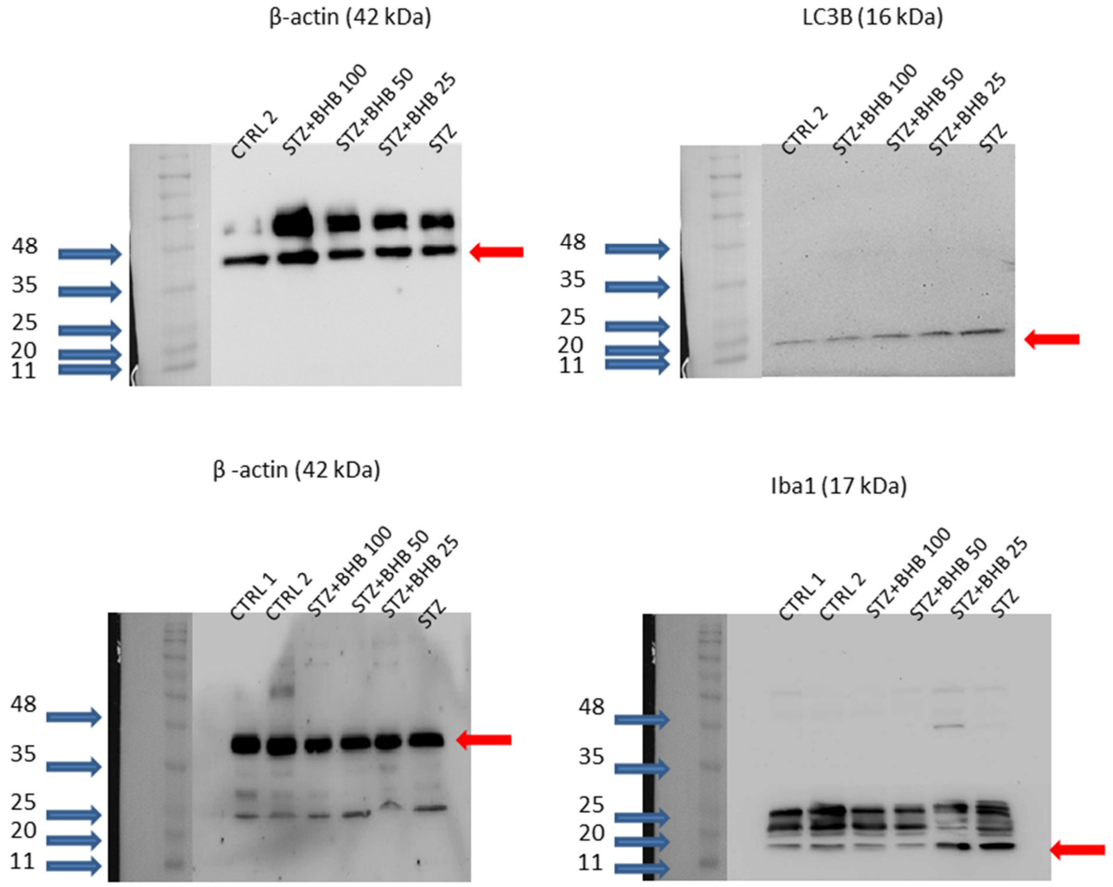

Figure S1. Uncropped images of representative β-actin, LC3B and Iba1 Western Blotting membranes.

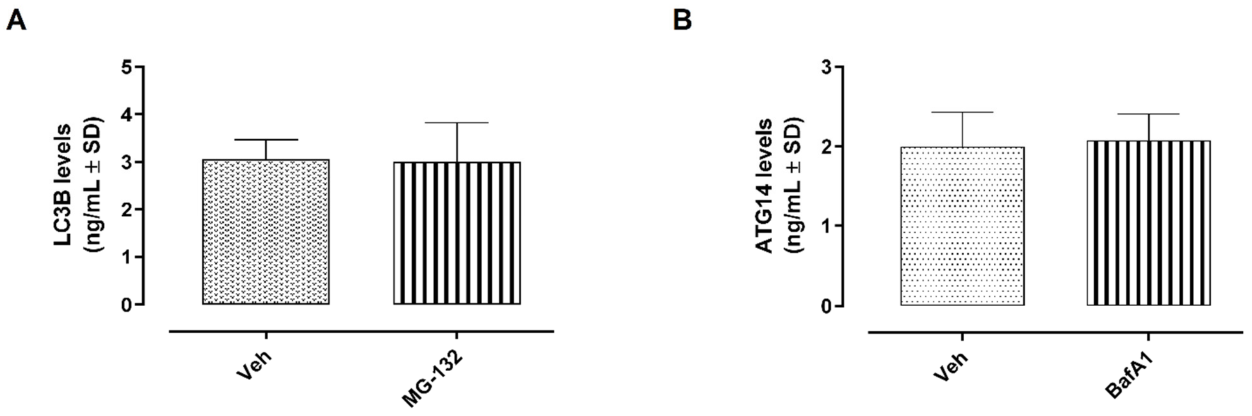

Figure S2. LC3B and ATG14 levels in STZ mice treated with MG-132 and bafilomicin A1. (A) LC3B levels, detected by ELISA Kit (ABIN6957822; Biocompare, Milan, Italy) after 2 weeks from contemporaneous i.p injections of STZ (75 mg/kg, single administration) and vehicle (Veh group; 0.5% DMSO) or STZ and MG-132 (MG-132 group; 10 μg/kg daily for 2 weeks). **(B)** ATG14 levels, detected by ELISA Kit (abx503511; abbexa, Cambridge, UK) after 2 weeks from contemporaneous i.p injections of STZ and vehicle (Veh group; 0.5% DMSO) or STZ and bafilomicin A1 (BafA1 group; 0.3 mg/kg daily, for 3 days).
